# Supplementary material for: Network-based proactive contact tracing: A pre-emptive, degree-based alerting framework for privacy-preserving COVID-19 apps
Source: PLOS Digit Health. 2025 Nov 19;4(11):e0000966. doi: 10.1371/journal.pdig.0000966 (PMC12629462; doi:10.1371/journal.pdig.0000966)
Supplement: S1 Appendix — Full list of model parameters and variables, including simulation, network, epidemic, and intervention-related quantities. (PDF) [file pdig.0000966.s001.pdf]

S1 Appendix. Nomenclature table. Full list of model parameters and variables, including simulation, network, epidemic, and intervention-related quantities. See Table A.

Table A. Complete list of parameters and variables.

**Symbol**	**Meaning**
Simulation & network parameters	
G_t	temporal contact network at time step t
ℓ	temporal snapshot window length (one hour)
$k_i(t)$	degree of node i in G_t
t	discrete time index (hourly snapshots)
T_j	intervention time j
Δt	intervention interval (time steps)
$N = \|V\|$	total number of nodes (population size)
β	per-edge transmission probability
γ	per-node recovery probability
Risk modeling	
$r_i(T_j)$	raw risk score (temporal degree) of node i at T_j
$r_{\max}$	global maximum of r_i over all snapshots of G_t
$\tilde{r}_i(T_j)$	normalized (log-scaled) risk score of node i at T_j
Epidemic indicators	
$I(t)$	number of infectious individuals at time t
$S(t)$	number of susceptible individuals at time t
$R(t)$	number of recovered individuals at time t
w	look-back window for $a(T_j)$
$a(T_j)$	clamped infection acceleration
$P(T_j)$	global infection potential
ω_a, ω_P	weights for a and P in ψ (both 1)
$\psi(T_j)$	epidemic-pressure signal
Threshold update	
$\theta(T_j)$	intervention threshold
λ	sensitivity of threshold to epidemic pressure
Intervention mechanism	
$\mathcal{H}(T_j)$	set of high-risk nodes
$X_i(t) \in \{S, I, R\}$	epidemic state of node i at time t
ϕ	fraction of edges removed for high-risk nodes
Cost–benefit metrics	
t_{peak}	time of peak prevalence in baseline
ΔI_{peak}	absolute reduction in peak prevalence
ΔI_{cum}	absolute reduction in cumulative infections
C_{edges}	cumulative percentage of edges removed
N_{HR}	total high-risk notifications sent
$\mathcal{E}_{\text{peak}}$	peak-efficiency ratio $\Delta I_{\text{peak}}/C_{\text{edges}}$
$\mathcal{E}_{\text{AR}}$	attack-rate efficiency $\Delta I_{\text{cum}}/C_{\text{edges}}$
