# Supplementary material for: Network-based proactive contact tracing: A pre-emptive, degree-based alerting framework for privacy-preserving COVID-19 apps
Source: PLOS Digit Health. 2025 Nov 19;4(11):e0000966. doi: 10.1371/journal.pdig.0000966 (PMC12629462; doi:10.1371/journal.pdig.0000966)
Supplement: S2 Appendix — Heatmaps and infection curves across varying β and γ for three networks: ABM, DTU, and Office. (PDF) [file pdig.0000966.s002.pdf]

S2 Appendix. Beta–Gamma sweeps. Heatmaps and infection curves across varying β and γ for three networks: ABM, DTU, and Office.

Fig A shows the exploration of the SIR parameter space for DTU, ABM and Office networks. Sweeping (β, γ)-pairs delimits the region of the plane that yields meaningful epidemic dynamics—avoiding trivial outcomes where the disease invariably dies out or infects the entire population—and guide our choice of transmission and recovery rates for the main experiments. We can observe a pronounced "phase-transition" band running diagonally across each heatmap: parameter combinations below this band correspond to outbreaks that fizzle out (final attack rates near 0 %), while those above it produce near-universal infection (final attack rates near 100 %).

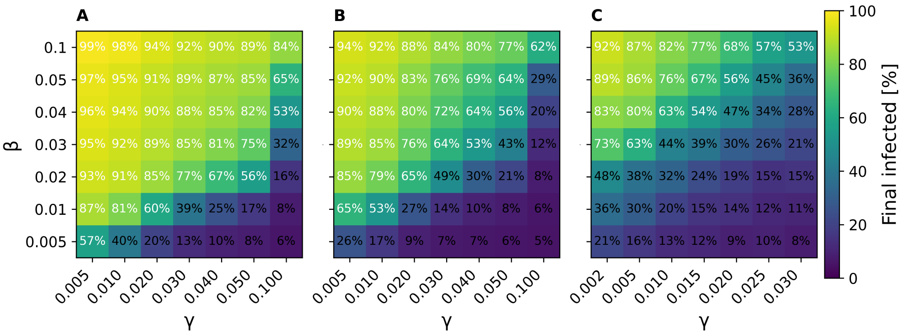

Fig A. Heatmaps of the final proportion of infected individuals over the (β, γ) parameter space. Results are shown for ABM (A), DTU (B), and Office (C). For each subplot, the vertical axis is the transmission rate β, the horizontal axis is the recovery rate γ, and the color intensity indicates the percentage of the population ultimately infected.

As described in the *SIR model* section, we require each SIR simulation to exhibit a pronounced infection peak and at least 50 % prevalence at peak, so that the impact of our NPCT interventions is clearly visible. In Fig B, we present additional (β, γ) pairs drawn from the band identified in Fig A, from which we selected one representative combination per network. We note that the Office network displays larger variance in its epidemic curves—reflecting its highly dynamic contact patterns—while the DTU network sometimes shows a monotonic rise with no clear peak before the 10-day horizon. This underscores the importance of examining both peak timing and overall outbreak size, rather than final attack rate alone, when evaluating intervention efficacy.

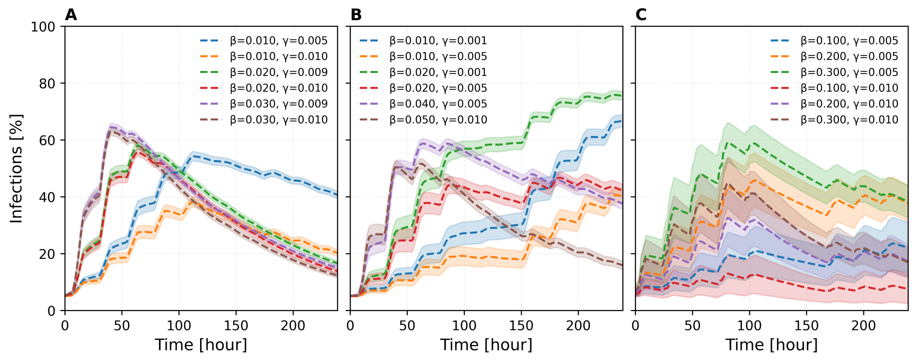

Fig B. Epidemic curves from (β, γ) parameter sweeps. Results are shown for ABM (A), DTU (B), and Office (C). Each subplot shows the time evolution of the percentage of infected individuals under varying transmission rate β and recovery rate γ. Shaded bands show ± 1 standard deviation.
