# Supplementary material for: Network-based proactive contact tracing: A pre-emptive, degree-based alerting framework for privacy-preserving COVID-19 apps
Source: PLOS Digit Health. 2025 Nov 19;4(11):e0000966. doi: 10.1371/journal.pdig.0000966 (PMC12629462; doi:10.1371/journal.pdig.0000966)
Supplement: S3 Appendix — Prevalence, edge count, and threshold tracking over time for the DTU and Office networks across different removal fractions ϕ. (PDF) [file pdig.0000966.s003.pdf]

**S3 Appendix. Epidemic dynamics across networks.** Prevalence, edge count, and threshold tracking over time for the DTU and Office networks across different removal fractions $\phi$.

For the DTU network (Fig A), the overall behavior closely mirrors what we observed on the ABM network. At low removal fractions ($\phi = 0.10, 0.25$), the reduction in peak prevalence remains modest, but once $\phi = 0.50$ is reached, a clear flattening of the infection curve appears for all sensitivity values $\lambda$. The adaptive threshold $\theta(T_j)$ likewise follows the same characteristic trajectory seen in the ABM case, with increasingly pronounced oscillations at higher removal levels. These findings show that NPCT interventions are effective at slowing and attenuating outbreaks in a range of network types, but the specific temporal patterns and contact heterogeneity critically shape their impact—making it essential to tailor hyperparameters like the removal fraction $\phi$ and sensitivity $\lambda$ to the network's dynamic characteristics.

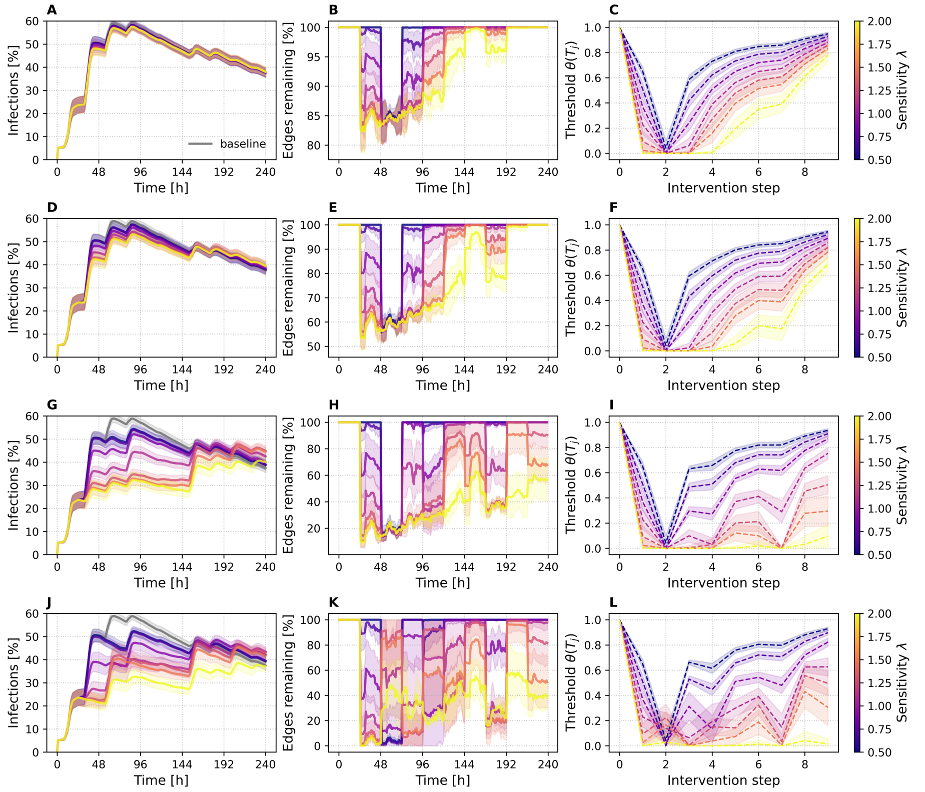


**Fig A. Progression of key quantities under NPCT interventions for DTU.** (A, D, G, J) Infection prevalence over time (hours, x-axis; percentage infected, y-axis), with the gray curve representing the no-intervention baseline. (B, E, H, K) Percentage of edges remaining in the contact network (relative to baseline) over time (hours). (C, F, I, L) Evolution of the adaptive threshold $\theta(T_j)$ across intervention steps. Colored curves represent sensitivity $\lambda \in [0.5, 2]$, results are averaged over SIR runs. Each row corresponds to a removal fraction $\phi \in \{0.10, 0.25, 0.50, 1.00\}$ (top to bottom). Shaded bands show $\pm 1$ standard deviation across 200 SIR runs.

The Office network (Fig B) also exhibits a flattened epidemic curve under NPCT, but with an additional signature of its temporal structure: the middle panels tracing the fraction of remaining edges regularly jump back to 100% during off-hours, when no workplace contacts are recorded.

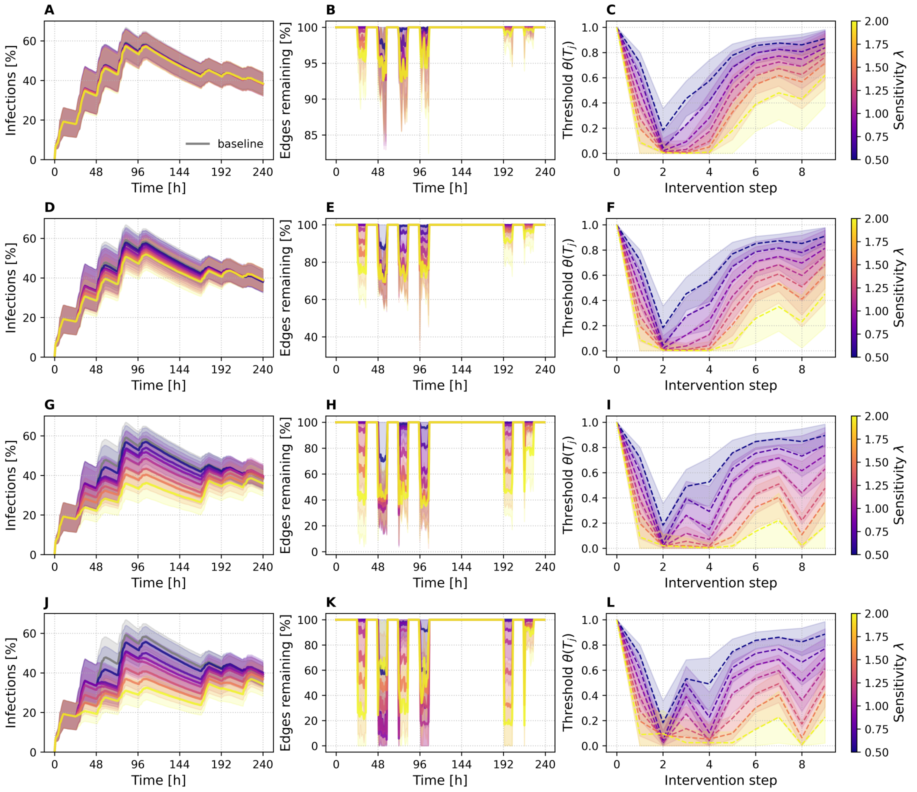


**Fig B. Progression of key quantities under NPCT interventions for Office.** (A, D, G, J) Infection prevalence over time (hours, x-axis; percentage infected, y-axis), with the gray curve representing the no-intervention baseline. (B, E, H, K) Percentage of edges remaining in the contact network (relative to baseline) over time (hours). (C, F, I, L) Evolution of the adaptive threshold $\theta(T_j)$ across intervention steps. Colored curves represent sensitivity $\lambda \in [0.5, 2]$, results are averaged over SIR runs. Each row corresponds to a removal fraction $\phi \in \{0.10, 0.25, 0.50, 1.00\}$ (top to bottom). Shaded bands show $\pm 1$ standard deviation across 200 SIR runs.
