# Supplementary material for: Network-based proactive contact tracing: A pre-emptive, degree-based alerting framework for privacy-preserving COVID-19 apps
Source: PLOS Digit Health. 2025 Nov 19;4(11):e0000966. doi: 10.1371/journal.pdig.0000966 (PMC12629462; doi:10.1371/journal.pdig.0000966)
Supplement: S4 Appendix — Concentration plots showing the burden of edge removals vs. cumulative population share (nodes ordered by increasing risk r~), stratified by ϕ and network type. (PDF) [file pdig.0000966.s004.pdf]

S4 Appendix. Burden distribution curves. Concentration plots showing the burden of edge removals vs. cumulative population share (nodes ordered by increasing risk $\tilde{r}$), stratified by ϕ and network type.

As shown in Fig A, we have reproduced the concentration curves for three removal fractions ($\phi = 0.10, 0.50, 1.00$) across the ABM, DTU, and Office networks. Consistent with the $\phi = 0.25$ results, the curves for different sensitivity values λ remain almost indistinguishable, confirming that varying λ has minimal impact on how the removal burden is distributed by risk. A slight exception occurs at $\phi = 1.00$ in the DTU and Office networks, where the $\lambda = 2.0$ curve shifts marginally closer to the equality line $y = x$, indicating a small move toward a more uniform workload. In the Office network panels, the curves also appear somewhat "stepped" rather than smooth, reflecting the smaller number of nodes: each individual removal constitutes a larger jump in the cumulative share, producing discrete increments. Even under full quarantine ($\phi = 1.00$), however, the choice of sensitivity parameter does not substantially alter the overall fairness of the intervention.

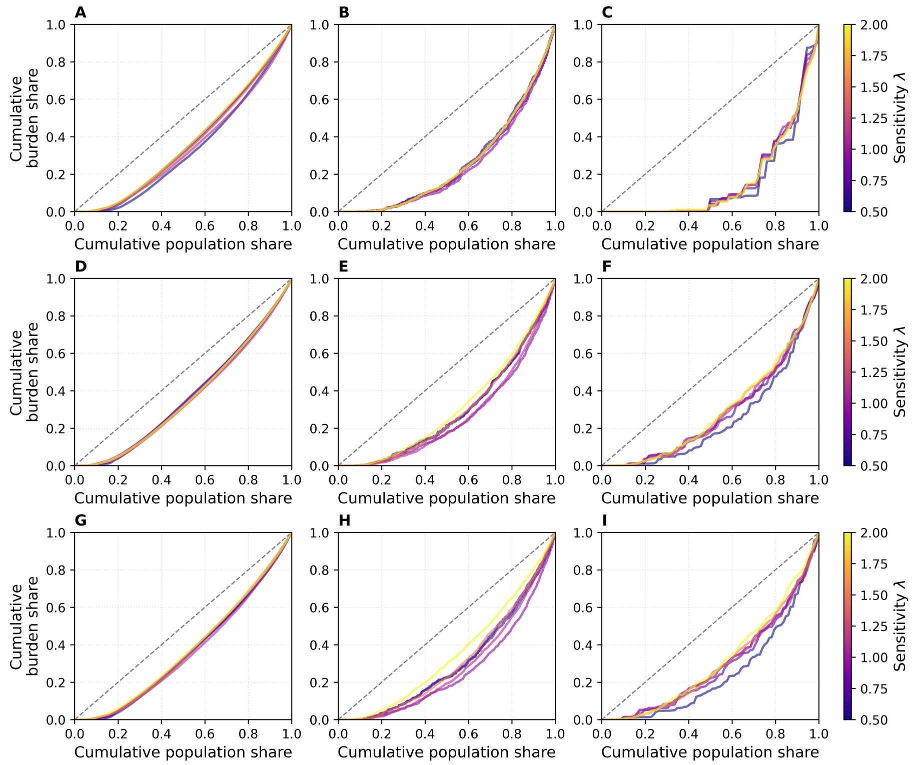

Fig A. Concentration curves of cumulative removal burden vs. cumulative population share (nodes ordered by increasing risk $\tilde{r}$) for removal fractions $\phi = 0.10, 0.50, 1.00$ across the ABM, DTU, and Office networks. The gray dashed line in each panel indicates perfect equality ($y = x$).
