# Supplementary material for: Network-based proactive contact tracing: A pre-emptive, degree-based alerting framework for privacy-preserving COVID-19 apps
Source: PLOS Digit Health. 2025 Nov 19;4(11):e0000966. doi: 10.1371/journal.pdig.0000966 (PMC12629462; doi:10.1371/journal.pdig.0000966)
Supplement: S5 Appendix — Risk histogram, ECDF of node selections, and alert burden over time for the Office network. (PDF) [file pdig.0000966.s005.pdf]

S5 Appendix. Risk and intervention distribution diagnostics. Risk histogram, ECDF of node selections, and alert burden over time for the Office network.

In Fig A, we show for the Office network three diagnostics—risk distribution, selection ECDF, and alert-burden time series. We use the 5th intervention ($\Delta t = 4$) and $\phi = 0.50$. While the risk distribution for the Office network is skewed toward lower $\tilde{r}$ values compared to the DTU and ABM networks, the ECDF of node selections reveals a markedly broader spread of total selections per node, especially for higher removal fractions.

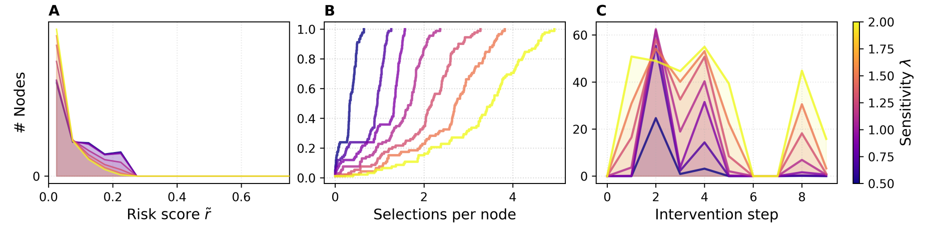

Fig A. Risk and alert characteristics under NPCT for Office network. (A) Histogram of per-node risk scores $\tilde{r}$ at intervention steps $\Delta t = 4$ ($\phi = 0.5$). (B) ECDF of total selections per node across ten interventions for $\phi = 0.5$. (C) Number of alerted nodes at each step for $\phi = 0.50$. Colors represent sensitivity λ, and results are averaged over SIR runs.
