# Supplementary material for: Network-based proactive contact tracing: A pre-emptive, degree-based alerting framework for privacy-preserving COVID-19 apps
Source: PLOS Digit Health. 2025 Nov 19;4(11):e0000966. doi: 10.1371/journal.pdig.0000966 (PMC12629462; doi:10.1371/journal.pdig.0000966)
Supplement: S6 Appendix — (PDF) [file pdig.0000966.s006.pdf]

S6 Appendix. Structural efficiency via fragmentation of the largest connected component. We evaluate whether NPCT preferentially removes structurally important edges by measuring, for each calendar day q, how much the largest connected component (LCC) of the *daily aggregated* contact network shrinks when the edges selected by NPCT are deleted. Let G_q denote the graph obtained by aggregating all contacts that occurred on day q. We define $\mathrm{LCC}_q^{\mathrm{pre}}$ as the size (number of nodes) of the largest connected component of G_q before any deletions. Let E_q^{NPCT} be the set of edges that NPCT removes on day q under the given sensitivity λ and removal fraction ϕ; after deleting these edges from G_q, the resulting LCC size is $\mathrm{LCC}^{\mathrm{post}}_{\mathrm{NPCT},q}$. For a random-removal baseline, we delete the same number of edges as $|E_q^{\mathrm{NPCT}}|$ but choose them uniformly at random; averaging over random draws yields $\mathrm{LCC}^{\mathrm{post}}_{\mathrm{rand},q}$. We aggregate contacts to daily graphs because interventions are applied once per day, aligning our structural measurement with the intervention cadence and keeping comparisons equivalent across days.

We report the daily LCC reductions

$$\Delta\mathrm{LCC}_{\mathrm{NPCT},q} = \mathrm{LCC}_q^{\mathrm{pre}} - \mathrm{LCC}^{\mathrm{post}}_{\mathrm{NPCT},q}, \qquad \Delta\mathrm{LCC}_{\mathrm{rand},q} = \mathrm{LCC}_q^{\mathrm{pre}} - \mathrm{LCC}^{\mathrm{post}}_{\mathrm{rand},q},$$

and the structural *lift* $\mathrm{lift}_q = \Delta\mathrm{LCC}_{\mathrm{NPCT},q}/\Delta\mathrm{LCC}_{\mathrm{rand},q}$. All values are means over SIR replicates (and, for the baseline, over random draws).

Table A. LCC fragmentation under NPCT vs. random removal. Means over replicates; "—" indicates undefined lift when $\Delta\mathrm{LCC}_{\mathrm{rand}} = 0$. Parameter settings per dataset: ABM ($\lambda = 1.0,\ \phi = 0.25$); DTU ($\lambda = 1.0,\ \phi = 0.5$); Office ($\lambda = 1.0,\ \phi = 0.5$).

Network	Day q	$\mathrm{LCC}^{\mathrm{pre}}$	$\mathrm{LCC}^{\mathrm{post}}_{\mathrm{NPCT}}$	$\mathrm{LCC}^{\mathrm{post}}_{\mathrm{rand}}$	$\Delta\mathrm{LCC}_{\mathrm{NPCT}}$	$\Delta\mathrm{LCC}_{\mathrm{rand}}$	Lift
ABM	1	1925.00	1920.12	1919.628	4.88	5.372	0.9084
ABM	2	1896.00	1702.90	1794.473	193.10	101.527	1.9020
ABM	3	1917.00	1737.60	1825.816	179.40	91.184	1.9675
ABM	4	1923.00	1754.00	1838.345	169.00	84.655	1.9963
ABM	5	1552.00	988.80	1394.587	563.20	157.413	3.5778
DTU	1	500.00	470.98	475.202	29.02	24.798	1.1703
DTU	2	486.00	341.58	375.867	144.42	110.133	1.3113
DTU	3	473.00	468.78	467.317	4.22	5.683	0.7426
DTU	4	267.00	141.56	141.589	125.44	125.411	1.0002
Office	1	73.00	72.92	72.926	0.08	0.074	1.0811
Office	2	59.00	21.90	18.416	37.10	40.584	0.9142
Office	3	70.00	65.58	66.022	4.42	3.978	1.1111
Office	4	62.00	28.64	29.996	33.36	32.004	1.0424

NPCT yields the strongest and most persistent fragmentation on the multi-location ABM network (e.g., day 5: $\Delta\mathrm{LCC} \approx 563.2$ versus 157.4 for random; lift ≈ 3.58), whereas DTU and Office show smaller or day-specific gains. This attenuation is expected: DTU and Office largely capture contacts within a single site, offering fewer inter-community bridges to break, while ABM embeds mobility-driven mixing across locations; removing high-risk edges therefore disrupts bridging paths more effectively and fragments the graph to a

greater extent. We report ABM results for days $q = 1$–5 and DTU/Office for $q = 1$–4; beyond these, under the chosen (λ, ϕ), NPCT selects no edges in DTU ($|E_q^{\mathrm{NPCT}}| = 0$) and the Office daily aggregate is essentially edgeless.
